# Trends of ARNI and Other Heart Failure Medication Use in Older Adults

**DOI:** 10.1093/geroni/igab046.805

**Published:** 2021-12-17

**Authors:** Yu-Chien Lee, Elisabetta Patorno, Chandrasekar Gopalakrishnan, Dae Kim

**Affiliations:** 1 Linkou Chang-Gung Memorial Hospital, Department of Family Medicine, Linkou Chang-Gung Memorial Hospital, Taoyuan, Taiwan (Republic of China); 2 Brigham and Women's Hospital and Harvard Medical School, Boston, Massachusetts, United States; 3 Hebrew SeniorLife, Boston, Massachusetts, United States

## Abstract

In 2015-2017, we identified 276,679 to 315,788 Medicare beneficiaries with HFrEF (mean age 76.6-76.7 years, 75.0-76.2% male, 82.0-83.4% Whites, and 44.8-50.9% frail). Since its approval in July 2015, ARNI use increased from 0.3% to 5.7%. ARNI uptake was lower in patients with older age (6.6% for 65-74 years vs 3.4% for ≥85 years), non-Hispanic race (7.3% for Hispanic vs 5.6-6.6% for other race), no dual eligibility (6.4% for dual eligibility vs 5.5% for no dual eligibility), frailty (5.1% for frailty vs 6.1% for non-frailty) and dementia (3.8% for dementia vs 6.1% for no dementia). Frail patients were less likely than non-frail patients to receive disease-modifying treatments, such as angiotensin-converting enzyme inhibitors (32.4% vs 38.9%), angiotensin receptor blockers (14.5% vs 17.5%), aldosterone antagonists (20.8% vs 23.4%), and beta-blockers (65.1% vs 68.3%), but more likely to receive symptomatic treatment with loop diuretics (56.4% vs 48.0%).

